# Survey on the impact of comorbid allergic rhinitis in patients with asthma

**DOI:** 10.1186/1471-2466-6-S1-S3

**Published:** 2006-11-30

**Authors:** Erkka Valovirta, Ruby Pawankar

**Affiliations:** 1 Turku Allergy Centre, 20610 Turku, Finland; 2 Nippon Medical School, Tokyo, Japan

## Abstract

**Background:**

Allergic rhinitis (AR) and asthma are inflammatory conditions of the airways that often occur concomitantly. This global survey was undertaken to understand patient perspectives regarding symptoms, treatments, and the impact on their well-being of comorbid AR and asthma.

**Methods:**

Survey participants were adults with asthma (*n *= 813) and parents of children with asthma (*n *= 806) from four countries each in the Asia-Pacific region and Europe. Patients included in the survey also had self-reported, concomitant AR symptoms. Patients and parents were recruited by telephone interview or by direct interview.

**Results:**

Most patients (73%) had pre-existing symptoms of AR when their asthma was first diagnosed. Shortness of breath (21%) was the most troublesome symptom for adults, and wheezing (17%) and coughing (17%) the most troublesome for children. Patients used different medications for treating asthma (most commonly short-acting β-agonists and inhaled corticosteroids) and for treating AR (most commonly oral antihistamines). The concomitant presence of AR and asthma disrupted the ability to get a good night's sleep (79%), to participate in leisure and sports activities (75%), to concentrate at work or school (69% of adults, 73% of children), and to enjoy social activities (57% of adults, 51% of children). Most patients (79%) reported worsening asthma symptoms when AR symptoms flared up. Many (56%) avoided the outdoors during the allergy season because of worsening asthma symptoms. Many (60%) indicated difficulty in effectively treating both conditions, and 72% were concerned about using excessive medication. In general, respondents from the Asia-Pacific region reported more disruption of activities caused by symptoms and more concerns and difficulties with medications than did those from Europe. Differences between the two regions in medication use included more common use of inhaled corticosteroids in Europe and more common use of Chinese herbal remedies in the Asia-Pacific region.

**Conclusion:**

Results of this survey suggest that comorbid asthma and AR substantially impact patient well-being and that the worsening of AR symptoms in patients with asthma can be associated with worsening asthma symptoms. These findings underscore the need for physicians who treat patients with asthma to evaluate treatment options for improving symptoms of both AR and asthma when present concomitantly.

## Background

Asthma and allergic rhinitis (AR) are chronic inflammatory conditions of the airways that affect a large proportion of the general population [1,2]. These diseases are often comorbid: 20–40% of patients with AR are reported to have asthma, and 30–90% of patients with asthma have AR [3-5]. AR is also a risk factor for developing asthma [3], and a number of studies have shown that AR usually precedes asthma in affected patients [6]. Moreover, the inflammatory mechanisms are similar in both conditions [7].

In addition to the symptomatic burden of AR in patients with asthma, comorbid AR may be associated with more asthma-related healthcare resource use, including emergency room visits and hospitalizations, and greater treatment costs than for patients with asthma alone [6,8-10]. The associations between AR and asthma with regard to epidemiology, to health-related outcomes, and to characteristics of inflammation are reviewed in the subsequent two papers in this supplement [11,12]. Our current understanding of the shared pathophysiology, health impairments, and socioeconomic costs of asthma and AR has culminated in the Allergic Rhinitis and its Impact on Asthma guidelines, which recommend a therapeutic 'one airway' approach that combines treatment of both upper and lower airways [2,3,13].

While there are several surveys assessing patient perspectives on asthma and on AR [14,15], relatively few have focused on the healthcare concerns and issues faced by patients burdened with both diseases [16]. The aim of this international survey of adult and pediatric patients in eight countries in Europe and Asia was to understand how and when the diagnoses of asthma and AR were made, to understand the most frequent and bothersome symptoms associated with asthma and AR, to understand medications used and attitudes toward such medications, and to understand the impact of comorbid asthma and AR on patient well-being.

## Methods

Adult patients 18–64 years of age and parents of patients 6–18 years of age were recruited for the study from four countries in the Asia-Pacific region (China, Singapore, South Korea, and Taiwan) and from four countries in Europe (France, Germany, Italy, and the United Kingdom). Households were randomly called, and potential respondents were screened to ensure recruitment of an equal number of adult patients and parents of pediatric patients with concomitant asthma and symptoms suggestive of AR. Potential respondents in the Asia-Pacific countries were also referred by other patients or intercepted at clinics, hospitals, and pharmacies and were interviewed face to face. A prior diagnosis of asthma and the presence of AR were self-reported by patients. AR was defined as congestion, sneezing, runny or itchy nose, and red, watery, itchy eyes.

The survey was conducted by an international research firm, which coordinated the translation of questions into the language of each country and collected and summarized the survey results. Telephone interviews lasted up to 15 minutes. Survey questions were not validated. The survey comprised 21 questions, some with multiple parts, investigating the onset and nature of allergic and asthmatic symptoms, the extent to which daily activities and sleep were affected by these symptoms, the types of allergy and asthma therapies, and the attitudes about these therapies.

## Results

### Study population

Survey participants included 809 patients from four countries in Europe and 810 patients from four countries in the Asia-Pacific region (Table 1). The average age of children was 11 years; 59% were male. The average age of adult patients was 40 years; 43% were male.

### Diagnosis

More than two-thirds of patients were already experiencing symptoms of AR at the time their asthma was diagnosed (Table 2). For a majority of patients (85%), the diagnosis of AR had been made by a healthcare provider, including a physician (81%), a Chinese herbalist (3%), or a pharmacist (1%). In the United Kingdom, the frequency of AR diagnosis was lower than in other countries: only 60% of patients reported a diagnosis of AR although they all reported symptoms of AR.

### Symptoms

A majority of patients (86% of adults and 88% of children) had experienced ≥5 symptoms identified in the survey. When the diagnosis of asthma was made at an earlier age (mean age 12.1 years), patients reported ≥9 symptoms of asthma and AR. By contrast, when the diagnosis was made later (age 15.6 years), patients reported fewer symptoms (≤5).

Overall, 73% of adults and 76% of children experienced cough at one time or another; 77% of adults and 81% of children experienced wheeze; 80% of adults and 79% of children experienced sneeze; 75% of adults and 79% of children experienced stuffy or blocked nose or nasal congestion; 77% of adults and 76% of children experienced a runny nose; 70% of adults and 72% of children experienced an itchy nose; and 61% of adults and 62% of children experienced red, watery, itchy, or puffy eyes.

The most frequently experienced symptoms and the most troublesome symptoms are presented by region and by age group in Table 3. In the Asia-Pacific region, almost one-half of patients (47%) experienced symptoms only during the allergy season. In the European region, 32% experienced symptoms only during the allergy season while 39% experienced symptoms throughout the year but more so during the allergy season (Table 3).

### Link between allergy and asthma

Almost two-thirds of patients (64%) believed that people with AR were, in general, also likely to have asthma. This was supported by the fact that a majority (69% of adults and 76% of children) had pre-existing symptoms of AR when asthma was first diagnosed. In this subgroup of patients with AR, however, only 43% had concerns (versus 51% with no concerns) about developing asthma before the diagnosis, although more parents were concerned about their children than adults were about themselves (55% versus 31%; *P *≤ 0.05).

### Asthma and allergy treatments

**Figure 1:**
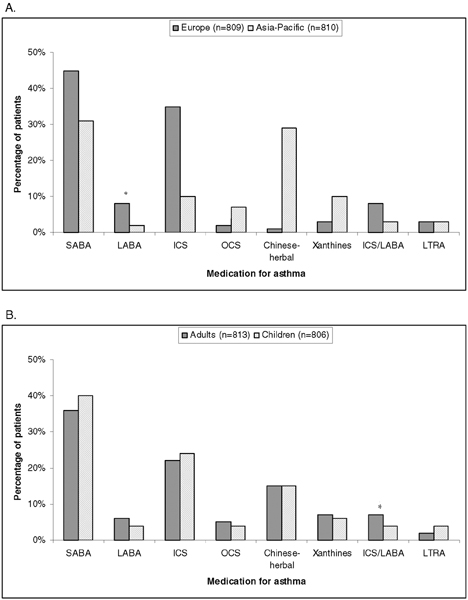
**Medications used for asthma**. Medications used by region (a) and by age group (b). *P ≤ 0.05 for comparison between Europe and the Asia-Pacific region or between adults and children. SABA, short-acting β-agonist; LABA, long-acting β-agonist; ICS, inhaled corticosteroid; OCS, oral corticosteroids; LTRA, leukotriene receptor antagonist.

The proportions of patients using asthma medications were significantly different (*P *≤ 0.05) between the European and Asia-Pacific regions for each category of asthma medication except leukotriene receptor antagonists, which were used by 3% of patients in each region (Figure 1). Short-acting β_2_-agonists were the most commonly used medications for asthma (38% of patients overall). A majority of patients on asthma medications (84% of adults and 85% of children) reported that they were very or fairly satisfied with their asthma treatment. Fewer parents of children than adults (77% versus 93%, *P *≤ 0.05) were very satisfied or fairly satisfied with inhaled corticosteroids or long-acting β-agonists.

**Figure 2:**
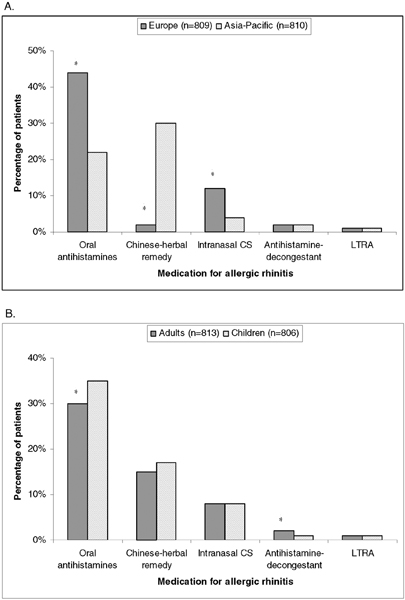
**Medications used for allergic rhinitis**. Medications used by region (a) and by age group (b). *P ≤ 0.05 for comparison between Europe and the Asia-Pacific region or between adults and children. CS, corticosteroid; LTRA, leukotriene receptor antagonist.

The percentages of patients using most categories of allergy medications were significantly different (*P *≤ 0.05) between the European and Asia-Pacific regions; antihistamines and nasal corticosteroids were used by more (*P *≤ 0.05) patients in Europe than in the Asia-Pacific region (Figure 2). Adults were less likely (*P *≤ 0.05) than children to use medications for their AR (77% versus 85%). Among those who used corticosteroids for their AR, 73% of adults and 80% of children were satisfied (very satisfied or fairly satisfied) with the treatment. Among antihistamine users, 77% of adults and 84% of children were satisfied.

Nevertheless, many patients (81% of adults and 82% of parents of children) were very interested or fairly interested in a single medication for treating both asthma and AR. The major reasons described by patients for this interest were as follows (with some overlapping values): 'easier to use' (36%), 'convenient' (26%), 'one tablet covers everything' (25%), 'less medication' (24%), and 'fewer side effects' (5%).

### Attitudes toward treatments

Attitudes about medications for asthma and AR are summarized in Table 4 by region and by age category. The percentages of patients agreeing with each survey question were significantly different between the European and Asia-Pacific regions, with typically greater percentages of patients in the Asia-Pacific region expressing difficulties or concerns about medications (see Table 4).

Overall, most patients (75%) agreed that asthma and AR bothered them in their day-to-day life. Moreover, most patients (60%) agreed that it was difficult to effectively treat both asthma and allergy at the same time and were concerned about using too much medication for both asthma and allergies (72%; see Table 4). Over three-quarters of patients were concerned, strongly or to some extent, about the potential side effects of corticosteroids (81% of adults and 88% of parents of children) for treating asthma and AR; these concerns were expressed regardless of whether patients were or were not already receiving corticosteroids (85% versus 84%, respectively) for one or both diseases. Sixty-four percent of adults and 67% of parents were concerned that their allergy medicine might make them or their children drowsy. Most patients agreed strongly or to some extent that they found it inconvenient to take different medications for concomitant asthma and allergy (63%), that nasal sprays were uncomfortable and/or unpleasant (58%), and that an oral medication was preferable to a nasal spray (60%) (see Table 4).

### Impact on well-being

**Figure 3:**
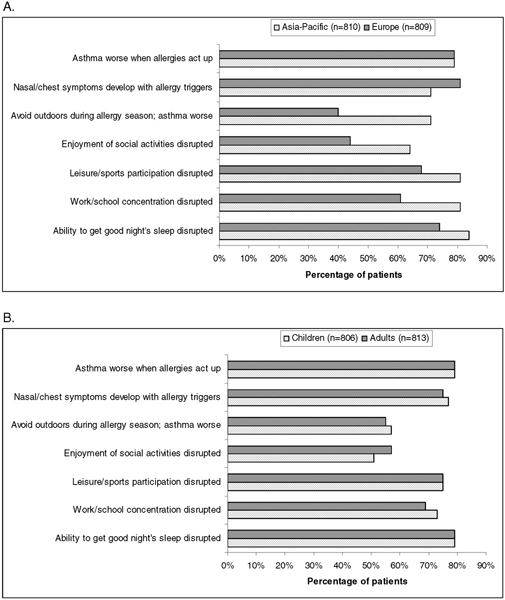
**Impact on patient well-being**. Frequency of symptoms and disruption of sleep and activities caused by comorbid asthma and allergic rhinitis, by region (a) and by age group (b). *P ≤ 0.05 for comparison between Europe and the Asia-Pacific region or between adults and children. For symptoms, a positive response was defined as symptoms occurring all of the time, a lot of the time, or some of the time. Disruption was defined as occurring a great deal, quite a lot, or a little bit.

A majority of patients reported disruptions in their sleep or daily activities ('a great deal,' 'quite a lot,' or 'a little bit') as a result of asthma and AR (Figure 3). Patients in the Asia-Pacific region reported most types of disruption significantly more commonly (*P *≤ 0.05) than those in the European region (Figure 3).

Among the overall survey population, comorbid asthma and AR disrupted the ability to get a good night's sleep (79%), to concentrate at work and school (71%), to participate in leisure and sports (75%), and to enjoy social activities (54%). Even among those reporting satisfaction ('a great deal,' 'quite a bit,' or 'a little bit') with their treatment for asthma, 52–78% reported an impairment in their well-being, with most (78%) reporting disruption in their ability to get a good night's sleep. Similarly, among those expressing satisfaction with their treatment for AR, 54–80% reported impairments, with most (80%) reporting disruption in their ability to get a good night's sleep. Among adults, 25–34% in each country believed that asthma and AR were equally disruptive to their well-being; parents of 30–35% of children believed the same. By country within region, 28–29% of patients in the European countries and 27–39% of patients in the Asia-Pacific region believed that asthma and AR were equally disruptive to their well-being.

Most patients reported that the symptoms of AR affected their asthma and their well-being during the allergy season all of the time, much of the time, or some of the time (Figure 3). Fifty-six percent avoided the outdoors during the allergy season because their asthma symptoms worsened when they were outside; 76% reported that nasal and chest symptoms developed with exposure to allergy triggers; and 79% reported that when their allergies acted up, their asthma symptoms became worse.

## Discussion

The results of this survey highlight the limitations imposed on daily life by the presence of comorbid asthma and AR. Approximately 70% or more of all patients reported a disruption in daily activities, including the ability to get a good night's sleep, to participate in leisure and sports activities, and to concentrate at work or school. More than one-half of patients reported that their symptoms had a negative affect on their ability to enjoy social activities.

More than two-thirds of the patients included in this survey were already experiencing AR symptoms when the diagnosis of asthma was made; this result reflects the comorbid nature of these disorders. For three-quarters of adult and pediatric patients, asthma symptoms increased some to all of the time when symptoms of AR flared up; similar numbers reported developing nasal and chest symptoms when coming into contact with allergy triggers. Interestingly, a recent small, prospective cohort study found that patients with concomitant asthma and AR experienced significantly worse physical functioning at pollen peak than those with only AR [17]. In the present study, over one-half of adult and pediatric patients avoided the outdoors during the allergy season because of fears that their asthma symptoms might worsen.

Some differences were seen between the two geographic regions in the frequency of asthma and AR symptoms, the bothersome nature of these symptoms, and the extent and duration of symptoms. In particular, the disruption of activities caused by symptoms was reported more commonly by respondents in the Asia-Pacific region than in Europe. Seventy-one percent of Asian respondents reported avoiding the outdoors during the allergy season because of fears that their asthma symptoms might worsen, while over 80% reported disruption of sleep, of concentration at work or school, and of participation in leisure or sports activities. Moreover, higher percentages of respondents from the Asia-Pacific region than from Europe reported concerns and difficulties with medications, including concerns about potential side effects of corticosteroids and about using too much medication for their asthma and allergies. Possible reasons for these differences between the two geographic groups of respondents could be cultural differences or differences between the two regions in the environment, allergic triggers, or treatments used.

Differences between the two regions were seen also in medication use by patients. In Europe, most patients received short-acting β_2_-agonists and inhaled corticosteroids for their asthma and received oral antihistamines for AR. By contrast, patients in the Asia-Pacific region used these treatments, particularly inhaled corticosteroids, less frequently and instead relied more on Chinese herbal remedies for both their asthma and their AR symptoms. It has been recently reported that such nonconventional treatments may have some benefit [18]. The methods used to study complementary and alternative therapies in rhinitis and asthma are poor, however, and in general there is little proof of efficacy for such therapies in these conditions [19].

It would have been interesting to know to what degree AR in and of itself contributed to decreased well-being. Among patients consulting with general practitioners for their AR in one study, 40% of those with mild rhinitis and >80% of those with moderate/severe rhinitis reported impaired activities, including impaired quality of life, sleep, daily activities, and work performance [20]. Distinguishing the relative contributions of asthma and AR to decreased well-being in the present survey would have been difficult, however – in part because all patients in the study had both conditions.

An inability to treat both asthma and AR together was a concern of respondents to the survey: approximately 60% of patients in each age group found it difficult to treat both conditions effectively at the same time. Moreover, two-thirds of adult patients and three-quarters of parents of pediatric patients were concerned about using too much medication to treat the two conditions.

The route of administration was an important consideration among survey respondents. Nearly 60% of all patients reported that they found nasal sprays uncomfortable or unpleasant for treating AR; and over one-half of all patients reported that they preferred using an oral medication to nasal sprays for treating these symptoms.

Patients' negative perceptions about side effects were also highlighted in the survey. Most respondents were concerned about the potential side effects of using corticosteroids for treating asthma or AR, and a majority of adult patients and parents were concerned that the allergy medicine might make them or their children drowsy.

A limitation of this survey is that participants were identified according to self-reports of asthma and symptoms of AR. Moreover, the survey questionnaire was not a validated instrument. In addition, patients' and parents' answers to questions about the impact of asthma and AR on well-being could be subject to recall bias.

A strength of this study is the large sample size (800 patients in Europe and 800 in the Asia-Pacific region). The representativeness and comparability of the two groups of participants is not certain, however, because survey participation in Europe was limited to individuals with a telephone in the household, whereas in Asia-Pacific countries some patients were recruited by other patients or at clinics. A combination of telephone and door-to-door surveys has been used by other surveys to evaluate asthma, including the Asthma Insights and Reality study [21].

## Conclusion

As highlighted by the Allergic Rhinitis and its Impact on Asthma guidelines [2], asthma and AR are related conditions and should be considered together when treatment options are discussed with patients. The results of this survey suggest that the worsening of AR symptoms in patients with asthma can be associated with worsening asthma symptoms, and that comorbid asthma and AR can cause substantial disruption in daily activities. Moreover, study respondents expressed concerns and difficulties with medications to treat asthma and AR.

AR and asthma are most commonly managed in the primary care setting. Physicians treating patients with asthma or AR must remain vigilant to the possible presence of the other condition, be aware of the risks posed by one condition to the development of the other, and evaluate treatment options for improving symptoms of both conditions when present concomitantly. In addition, physicians must be aware of possible patient concerns about medications, particularly patient concerns about potential side effects of corticosteroids and about using too much medication for their asthma and allergies. More generally, there is a need to promote the use of combined therapies that are safe and effective for treating symptoms of both asthma and AR, and that address the inflammatory nature of these two conditions affecting the 'one airway.'

## Abbreviations

AR = allergic rhinitis.

## Competing interests

EV has received speaker's honoraria from Merck & Co. RP declares that she has no competing interests.

## Figures and Tables

**Table 1:** Study populations

Country and region	Number of adults			Number of children			Total
		
	Total	18–34 years^a^	35 to ≥65 years^b^	Total	6–14 years	15–18 years^a^	
Europe							
France	102	39	63	100	59	41	202
Germany	102	40	62	100	80	20	202
Italy	104	45	59	100	70	30	204
United Kingdom	101	32	69	100	70	30	201
Total	409	156	253	400	279	121	809
Asia-Pacific							
China	100	73	60	100	74	26	200
Singapore	100	40	27	100	79	21	200
South Korea	100	11	89	100	91	8	200
Taiwan	104	44	60	106	94	12	210
Total	404	168	236	406	338	68	810
Global total	813	324	489	806	617	189	1619

^a^Because of the way the questions were asked, 18-year-old patients (20 in Europe, one in Asia-Pacific) could have been included in either the adult or the pediatric populations.

^b^The only patients ≥65 were from South Korea (*n *= 17).

**Table 2:** Diagnosis of asthma and allergic rhinitis (AR)

	Europe (*n *= 809)	Asia-Pacific region (*n *= 810)	Adults (*n *= 813)	Children (*n *= 806)
Mean age (years) when asthma was diagnosed	13.2	13.2	21.2*	5.1
Mean age (years) when AR was diagnosed	13.5	13.3	21.7*	5.5
Percentage of patients already experiencing AR symptoms at the time of diagnosis of asthma	69*	77	69*	76
Percentage of patients who had diagnosis of AR				
By a physician	84*	78	77*	84
By a pharmacist	1	2	1	1
By a Chinese herbalist	0*	6	4	2
Not diagnosed by healthcare provider	15	15	18*	13

*Significant (*P *≤ 0.05) difference between Europe and the Asia-Pacific region or between adults and children.

**Table 3:** Most frequent or troublesome symptoms and their occurrence

	Percentage of patients			
	
	Europe (*n *= 809)	Asia-Pacific region (*n *= 810)	Adults (*n *= 813)	Children (*n *= 806)
Most frequently experienced symptoms				
Stuffy, blocked nose or congestion	26*	47	34*	39
Sneezing	32*	47	38	42
Red, watery, itchy, or puffy eyes	37*	20	27	30
Coughing	23*	40	27*	37
Most troublesome symptoms				
Stuffy, blocked nose or congestion	10	13*	13	12
Red, watery, itchy, or puffy eyes	16*	4	10	10
Wheezing	17	16	16	17
Shortness of breath	18	17	21*	15
Coughing	9*	19	11*	17
Extent and duration of symptoms				
Just during allergy season	32*	47	40	39
Consistently throughout year	27*	23	27*	23
Throughout year, but more during season	39*	28	31*	36

*Significant (*P *≤ 0.05) difference between Europe and the Asia-Pacific region or between adults and children.

**Table 4:** Attitudes of survey participants about asthma and allergic rhinitis medications

Attitude expressed	Percentage of patients in agreement with survey question^a^			
	
	Europe (*n *= 809)	Asia-Pacific region (*n *= 810)	Adults (*n *= 813)	Children (*n *= 806)
Asthma and/or allergies interfere with day-to-day life	70*	80	74	76
Difficult to treat asthma and allergy conditions effectively at the same time	47*	73	58	62
Concerned about using too much medication for both asthma and allergies	59*	85	65*	79
Concerned about the potential side effects of using corticosteroids for asthma and allergies	76*	92	81*	88
Concerned that an allergy medication that might cause drowsiness	54*	77	64	67
Inconvenient to take different medications for asthma and allergy symptoms	47*	78	63	62
Nasal sprays uncomfortable/unpleasant to treat allergies	52*	65	57	59
Oral medication preferable over nasal spray for allergies	62*	57	60	60

*Significant (*P *≤ 0.05) difference between Europe and the Asia-Pacific region or between adults and children.

^a^Agreement was defined as agree strongly or agree somewhat.

## References

[B1] National Institutes of Health NH Lung and Blood Institute (2002). Asthma management and prevention. Global Initiative for Asthma. A practical guide for public health officials and health care professionals. Based on the global strategy for asthma management and prevention NHLBI/WHO workshop report. Updated report 2002.

[B2] Bousquet J, Van Cauwenberge P, Khaltaev N (2001). Allergic rhinitis and its impact on asthma. J Allergy Clin Immunol.

[B3] Leynaert B, Neukirch F, Demoly P, Bousquet J (2000). Epidemiologic evidence for asthma and rhinitis comorbidity. J Allergy Clin Immunol.

[B4] Simons FE (1999). Allergic rhinobronchitis: the asthma–allergic rhinitis link. J Allergy Clin Immunol.

[B5] Gaugris S, Sazonov-Kocevar V, Thomas M (2006). Burden of concomitant allergic rhinitis in adults with asthma. J Asthma.

[B6] Braunstahl GJ, Fokkens W (2003). Nasal involvement in allergic asthma. Allergy.

[B7] Pawankar R (2006). Allergic rhinitis and asthma: are they manifestations of one syndrome?. Clin Exp Allergy.

[B8] Price D, Zhang Q, Kocevar VS, Yin DD, Thomas M (2005). Effect of a concomitant diagnosis of allergic rhinitis on asthma-related health care use by adults. Clin Exp Allergy.

[B9] Thomas M, Kocevar VS, Zhang Q, Yin DD, Price D (2005). Asthma-related health care resource use among asthmatic children with and without concomitant allergic rhinitis. Pediatrics.

[B10] Sazonov Kocevar V, Thomas J, Jonsson L, Valovirta E, Kristensen F, Yin DD, Bisgaard H (2005). Association between allergic rhinitis and hospital resource use among asthmatic children in Norway. Allergy.

[B11] Thomas M (2006). Allergic rhinitis: evidence for impact on asthma. BMC Pulm Med.

[B12] Jeffery PK, Haahtela T (2006). Allergic rhinitis and asthma: inflammation in a one airway condition. BMC Pulm Med.

[B13] Pawankar R (2004). Allergic rhinitis and asthma: the link, the new ARIA classification and global approaches to treatment. Curr Opin Allergy Clin Immunol.

[B14] Jones KG, Bell J, Fehrenbach C, Pearce L, Grimley D, McCarthy TP (2002). Understanding patient perceptions of asthma: results of the Asthma Control and Expectations (ACE) survey. Int J Clin Pract.

[B15] Demoly P, Allaert FA, Lecasble M (2002). ERASM, a pharmacoepidemiologic survey on management of intermittent allergic rhinitis in every day general medical practice in France. Allergy.

[B16] Leynaert B, Neukirch C, Liard R, Bousquet J, Neukirch F (2000). Quality of life in allergic rhinitis and asthma. A population-based study of young adults. Am J Respir Crit Care Med.

[B17] Laforest L, Bousquet J, Pietri G, Sazonov Kocevar V, Yin D, Pacheco Y, Van Ganse E (2005). Quality of life during pollen season in patients with seasonal allergic rhinitis with or without asthma. Int Arch Allergy Immunol.

[B18] Brinkhaus B, Hummelsberger J, Kohnen R, Seufert J, Hempen CH, Leonhardy H, Nogel R, Joos S, Hahn E, Schuppan D (2004). Acupuncture and Chinese herbal medicine in the treatment of patients with seasonal allergic rhinitis: a randomized-controlled clinical trial. Allergy.

[B19] Passalacqua G, Compalati E, Schiappoli M, Senna G (2005). Complementary and alternative medicine for the treatment and diagnosis of asthma and allergic diseases. Monaldi Arch Chest Dis.

[B20] Bousquet J, Neukirch F, Bousquet PJ, Gehano P, Klossek JM, Le Gal M, Allaf B (2006). Severity and impairment of allergic rhinitis in patients consulting in primary care. J Allergy Clin Immunol.

[B21] Rabe KF, Adachi M, Lai CK, Soriano JB, Vermeire PA, Weiss KB, Weiss ST (2004). Worldwide severity and control of asthma in children and adults: the global asthma insights and reality surveys. J Allergy Clin Immunol.

